# Management of abdomen hydatidosis after rupture of a hydatid splenic cyst: a case report

**DOI:** 10.4076/1757-1626-2-8416

**Published:** 2009-09-02

**Authors:** Christos Limas, Chrisostomos Soultanidis, Michail A Kirmanidis, Christina Tsigalou, Savvas Deftereos

**Affiliations:** 1 Paediatric Surgery Department, University General Hospital of AlexandroupolisDragana, 68100 AlexandroupolisGreece; 2 Microbiology Laboratory, University General Hospital of AlexandroupolisDragana, 68100 AlexandroupolisGreece; 3 Radiology Department, University General Hospital of AlexandroupolisDragana, 68100 AlexandroupolisGreece

## Abstract

We present a case of multiple hydatidosis in an 8-year-old boy that resulted from a ruptured hydatid cyst of the spleen and spread into the peritoneal cavity. We also present a new approach for managing these difficult and high-risk cases.

## Introduction

Hydatidosis is a parasitic disease caused by *Echinococcus granulosus* in humans and many other mammals. The disease is characterized by a worldwide distribution but is endemic mainly around the Mediterranean Sea. There has been recently considerable progress in the diagnosis and management of hydatid disease. Although the radical therapy remains the surgical excision of the cyst, very good results have been reported by the administration of chemotherapy with anthelminthics (mainly albendazole). Preoperative administration of chemotherapy aims to sterilize the cysts, to soften them, and to reduce intracystic pressure enabling the surgeon to remove the endocyst more easily, whereas postoperatively is used to reduce the rate of recurrence.

## Case presentation

An 8-year-old boy, Greek, presented in the emergency department with abdominal pain, abdominal distension, diarrheas and fever. A physical examination revealed multiple palpable painless abdominal masses that were mainly confined to the lower abdomen and around the omphalic region. He had a history of acute abdominal pain and anaphylactic reaction with urticaria rash 2 months previously, but this had regressed without medical therapy.

**Figure 1.**
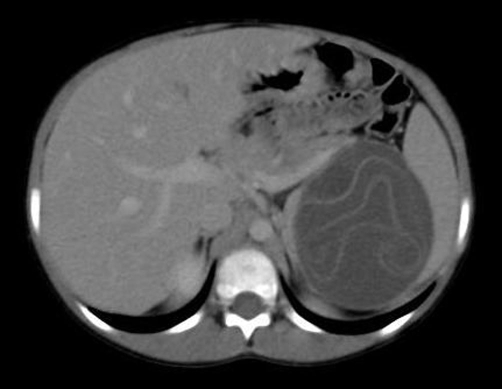
CT scan demonstrating floating membrane in the splenic cyst and multiple cysts in the peritoneal cavity.

**Figure 2.**
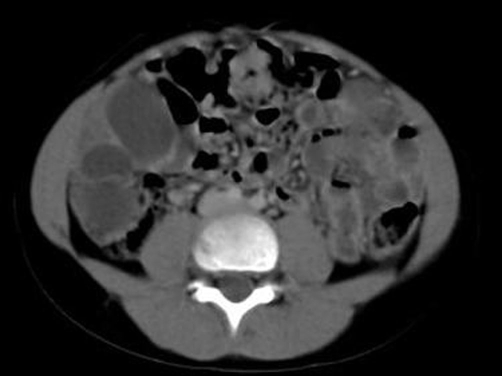
CT scan demonstrating floating membrane in the splenic cyst and multiple cysts in the peritoneal cavity.

An ultrasound and computed tomography examination revealed a cyst containing a “floating membrane” in the spleen and numerous unilocular cysts in the peritoneal cavity, measuring from a few millimeters to 3 cm (Figures 1 and 2) Floating membrane in the splenic cyst and multiple cysts in the peritoneal cavity). Laboratory data were within normal limits except for the eosinophil count (12%) and antibodies against *Echinococcus granulosus* (1/4096). The diagnosis of multiple hydatid disease was established based on imaging findings and positivity for anti-echinococcal antibodies.

We applied chemotherapy (albendazole, 10 mg/Kgr/day) for 6 months, since the spread of the disease into the peritoneal cavity and the multiplicity of lesions were strong contraindications for a radical surgical approach. At around 3 months the cysts in the peritoneal cavity had disappeared and the splenic cyst had reduced in size. However the echinococcal antibody titers, (which were measured monthly), did not fall noticeably throughout the 6-month chemotherapy period.

**Figure 3.**
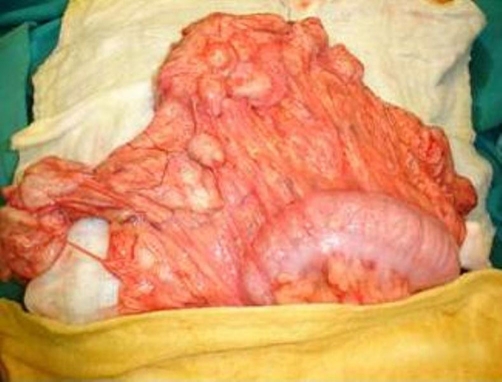
Numerous cysts in the great omentum.

Our fundamental concerns on exploration were to avoid spreading the disease and to preserve the spleen. A wide trocar was used to open the splenic cyst and suck the contents out. The result was negative since the contents appeared to be gelatinous rather than watery. The splenic cyst cleared and drained externally. The exploration of the abdominal cavity revealed a myriad of cysts, measuring from a few millimetres to 3 cm located only in the great omentum (Figure 3) numerous cysts in the great omentum). The surgical handlings was very easy and there was no possibility of spillage since the cysts were thick, tough, difficult to cut even with scissors, and containing only a yellowish gelatin like substance. The greater omentum was entirely removed, including all the cysts. The postoperative course was uneventful, and the child was discharged on the 7^th^ postoperative day. Six months later the child was well, with a slow decrease in titers and no sign of remaining disease.

## Discussion

Hydatid cysts compose of three layers: the pericyst, the laminated membrane and the endocyst (germinal layer). The cyst fluid is a transudate of serum that contains proteins and is antigenic [1]. The liver is the most commonly infected organ in the human body (55-70% of cases), followed by the lungs and spleen (20-35% of cases combined) [2]. Splenic hydatid cysts usually coexist with liver hydatid cysts, although in some cases the spleen is the primary location. The cysts typically remain silent for years, with their size and location determining the manifesting symptoms. The first symptoms are often caused by a complication, mainly rupture of the cyst due to trauma or increased intracystic pressure, which causes fever, urticaria, eosinophilia and anaphylactic shock, as well as spreading of the disease [3].

Immunodiagnostic tests lack specificity, but they can be helpful in the diagnosis since their sensitivity varies from 60% to 90%. Results of various serologic tests for echinococcus should always be interpreted in conjunction with the patient's medical history, clinical presentation and other findings. The reaction can be present even years after the surgical removal of the cyst, since blood titers decrease only slowly [1]. A serologic survey is critical in the follow-up of operated patients since a positive test during this period is not diagnostic of recurrence, whereas a rising titter is. Although serological tests are useful, abdominal ultrasonography and computed tomography are necessary to establish the diagnosis.

Surgical excision is still the only curative treatment, but good results have been reported with albendazole administration. Medical treatment is indicated in cysts inaccessible for surgical removal or as a complementary therapy to prevent recurrence [4]. Preoperative administration of anthelminthics sterilizes and softens the cysts, and reduces intracystic pressure, facilitating the removal of the endocyst and reducing the rate of recurrence [5]. This preoperative administration is reported to last from days to 2 months [6,7]. In our opinion a longer duration of preoperative anthelminthics administration, -up to 6 months-, will completely degenerate the cysts, facilitate surgical handlings, minimize the risk of spillage and make the exploration safer.
